# Organoboron‐Functionalization Enables the Hierarchical Assembly of Giant Polyoxometalate Nanocapsules

**DOI:** 10.1002/anie.202003550

**Published:** 2020-04-08

**Authors:** Shujun Li, Yanfang Zhou, Nana Ma, Jie Zhang, Zhiping Zheng, Carsten Streb, Xuenian Chen

**Affiliations:** ^1^ School of Chemistry and Chemical Engineering Henan Key Laboratory of Boron Chemistry and Advanced Energy Materials Henan Normal University Xinxiang Henan 453007 China; ^2^ Shenzhen Grubbs Institute and Department of Chemistry Southern University of Science and Technology Shenzhen Guangdong 518055 China; ^3^ Institute of Inorganic Chemistry I Ulm University Albert-Einstein-Allee 11 89081 Ulm Germany; ^4^ College of Chemistry and Molecular Engineering Zhengzhou University Zhengzhou 450001 China

**Keywords:** boronic acid, organo-functionalization, polyoxometalate, self-assembly, supramolecular chemistry

## Abstract

The aggregation of molecular metal oxides into larger superstructures can bridge the gap between molecular compounds and solid‐state materials. Here, we report that functionalization of polyoxotungstates with organo‐boron substituents leads to giant polyoxometalate‐based nanocapsules with dimensions of up to 4 nm. A “lock and key” mechanism enables the site‐specific anchoring of aromatic organo‐boronic acids to metal‐functionalized Dawson anions [M_3_P_2_W_15_O_62_]^9−^ (M=Ta^V^ or Nb^V^), resulting in unique nanocapsules containing up to twelve POM units. Experimental and theoretical studies provide initial insights into the role of the organo‐boron moieties and the metal‐functionalized POMs for the assembly of the giant aggregates. The study therefore lays the foundations for the design of organo‐POM‐based functional nanostructures.

## Graphical abstract

**Giants**: Polyoxometalate based tetrameric and dodecameric nanocapsules with diameters up to 4 nm are accessed in the solid state by targeted organo‐functionalization of metal‐functionalized Dawson anions with aromatic boronic acids.

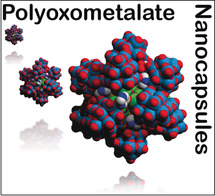

The assembly of molecular metal oxides, so‐called polyoxometalates (POMs), into nanostructured supramolecular architectures holds great promise for the bottom‐up design of molecular functional materials.^1^, ^2^, ^3^ POMs are molecular metal‐oxide anions whose properties can be tuned by chemical modification, enabling applications in catalysis, energy conversion, molecular electronics and materials science.^4^−^6^ POMs are ideal prototypes for aggregation into larger nanostructures,^7^, ^8^, ^9^ as a number of POM‐linkage concepts have been established: currently, the field is dominated by the linkage of POMs using metal cation (often transition metal or lanthanide) connectors.^10^ To‐date, this concept has been most often used in polyoxotungstate chemistry, where Keggin‐ and Dawson‐anions and their derivatives have been linked into giant cluster‐of‐cluster aggregates^11^, ^12^, ^13^, ^14^, ^15^ or even polyoxometalate‐based open frameworks.^2^, ^16^ One promising alternative is the linkage of POMs using organic functionalization;^17^, ^18^, ^19^, ^20^, ^21^ this concept has not been widely used in POM nanostructure design. However, pioneering studies have demonstrated its versatility. In a ground‐breaking study, Cronin and colleagues used Dawson‐polyoxotungstates functionalized with organic primary ammonium groups to aggregate tetrahedral nanostructures based on four Dawson anions using multiple hydrogen bonds between the organo‐ammonium groups and the cluster oxide surface. The authors also demonstrated the aggregate stability and dynamics in solution/gas phase by high‐resolution mass spectrometry.^22^ Yaghi and colleagues used organo‐functionalized Anderson molybdates as linear building units for the assembly of POM‐based covalently linked metal organic frameworks with high ionic conductivities.^23^ Izzet and colleagues have used Dawson anions functionalized with organic terpyridine side‐chains and demonstrated their linkage into supramolecular triangles and larger aggregates by coordination of Fe^2+^ to the terpyridine coordination sites.^24^

To‐date, most organo‐functionalization approaches use group 14 and group 15 elements to attach organic groups to POMs by forming M−O−X−R bonds (X=C, Si, Ge, Sn, P, As Sb).^17^, ^18^ Here, we suggest that boronic acids could be an easily accessible alternative for the facile functionalization of POMs with organic groups.^25^ To the best of our knowledge, covalent POM‐linkage using boronic acids has thus far not been reported.^17^, ^18^, ^21^ However, given the high oxophilicity and electron deficiency of boron, it could be well suited to access organoboron‐functionalized POMs.^26^ In addition, boronic acids are particularly well suited for designing supramolecular POM aggregates, as they are used in molecular nanostructures^27^, ^28^ and dynamic covalent chemistry.^29^

Here, we propose a new mechanism for the covalent functionalization of POMs with boronic acids. The concept combines the electron‐deficiency and electrophilicity of the boron atom in boronic acids^30^ with the nucleophilicity and high electron density of metal‐oxo groups in POMs. We hypothesized that tungstate clusters functionalized with the group 5 metals Nb^V^ and Ta^V^ would provide the ideal combination of structural/chemical stability and metal oxo bond polarization, as oxo ligands bound to Nb/Ta are significantly more nucleophilic compared with the classical Mo−O and W−O species, see Scheme 1.^31^, ^32^, ^33^ Here, we use this synthetic concept to connect multiple Dawson anions [M_3_P_2_W_15_O_62_]^9−^ (=**{M_3_W_15_}**, M=Nb, Ta) into POM‐based nanostructures using aromatic boronic acid linkages, resulting in POM‐aggregates with diameters of up to 4 nm, internal cavities and unique high‐symmetry architectures.

**Scheme 1:**
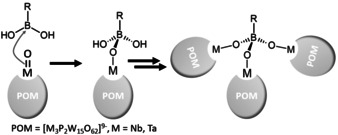
proposed mechanism for the boronic acid organo‐functionalization and linkage of Nb/Ta‐functionalized polyoxotungstates.

Briefly, the POM‐based nanostructures were synthesized by reaction of [(MO_2_)_3_P_2_W_15_O_59_]^9−^ (M=Ta, Nb) with the respective boronic acid (3‐pyridineboronic acid (=3‐PyBH_2_) or 5‐pyrimidinylboronic acid (5‐PymBH_2_)) in acidic aqueous solution at 75 °C. All compounds reported were isolated as single‐crystalline, pure products (full analyses see Supporting Information), enabling their characterization by single‐crystal X‐ray diffraction (crystallographic details see Supporting Information, Table S1).^34^ Table 1 shows a summary of the four compounds reported: Note that **1‐Ta** and **1‐Nb** are isostructural, the structural details are discussed using **1‐Ta** as example: **1‐Ta** crystallizes in the monoclinic space group *I*2/a with cell parameters *a=*26.9889(4) Å, *b=*49.6651(5) Å, *c=*32.2254(4) Å, *β*=105.7122(16)°, *V=*41 581.1(10) Å^3^.

**Table 1:** Boronic acid‐functionalized POMs obtained by reaction of [M_3_P_2_W_15_O_62_]^9−^ with 3‐PyBH_2_ or 5‐PymBH_2_.

**1**	{Na(3‐PyB)_4_[M_3_P_2_W_15_O_62_]_4_}^27−^, M=Ta (**1‐Ta**), Nb (**1‐Nb**)
**2**	{K_4_(5‐PymB)_3_(5‐PymBH)_12_[M_3_P_2_W_15_O_62_]_12_}^86−^, M=Ta (**2‐Ta**), Nb (**2‐Nb**)

In **1‐Ta**, four **{Ta_3_W_15_}** units are linked by four 3‐PyB moieties into a tetrahedral aggregate with maximum diameter of ≈2.8 nm (Figure 1 and Figure S1) and a molecular weight of ≈20 kDa. Each boron center of 3‐PyB acts as tritopic linker to three neighboring **{Ta_3_W_15_}** units by forming one B−O−Ta bond to the respective cluster, resulting in a stabilization of the aggregate by twelve B−O−Ta bonds. The boronic acids bind selectively to the tantalum sites as proposed in Scheme 1. In the center of **1‐Ta**, a central void with a diameter of ≈3 Å is observed which is occupied by an encapsulated Na^+^ ion (Figure 1 D/E). **1‐Ta** can therefore be described as a tetrahedron with edge‐length of 2.8 nm where each face is capped by the B atom of the 3‐PyB moieties (Figure 1 C).

**Figure 1:**
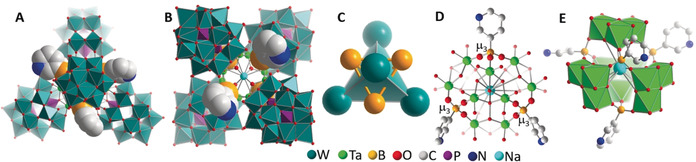
The tetrameric nanocapsule **1‐Ta. A**: view along the *C*_3_‐axis; **B**: view along the *C*_2_‐axis; **C**: the Dawson‐based tetrahedron (teal) and the face‐capping by the boronic acid boron atoms (orange); **D**: detailed ball‐and‐stick illustration of the central tantalum‐oxo‐boronic acid core; **E**: polyhedral view of the central core. Hydrogen atoms are omitted for clarity.

As second class of boronic‐acid‐linked nanocapsules, we report the isostructural compounds **2‐Ta** and **2‐Nb**; the following discussion is based on the crystal structure of **2‐Ta. 2‐Ta** crystallizes in the trigonal space group *R*‐3 with cell parameters *a*=*b=*57.3040(8) Å, *c=*65.4141(10) Å, *V=*18 605(6) Å^3^. In **2‐Ta**, dodecameric nanocapsules based on twelve **{Ta_3_W_15_}** units linked by 5‐PymB boronic acids are present, each capsule features an outer diameter of ≈4 nm, an inner cavity of ≈1.3 nm and a molecular weight of ≈57 kDa, see Figure 2, Figures S2–S4. The dodecameric capsules are based on trimeric building units **{Ta_3_W_15_}_3_**, where two types of boronic acid act as linkages: a central μ_3_‐bridging 5‐PymB unit links the three **{Ta_3_W_15_}** groups by three Ta−O−B bonds (Figure 2 B). In addition, three peripheral mono‐protonated 5‐PymBH units act as μ_2_‐bridges between two neighboring **{Ta_3_W_15_}** clusters (Figure 2 B), resulting in the trimers with idealized *C*_3*v*_ symmetry. Four **{Ta_3_W_15_}_3_** trimers are then linked into the final dodecameric **2‐Ta** structure (=[**{Ta_3_W_15_}_3_**]_**4**_) by coordination of K^+^ ions to adjacent cluster shells. Intriguingly, only three of the **{Ta_3_W_15_}_3_** units feature the central μ_3_‐bridging boronic acid, while the fourth **{Ta_3_W_15_}_3_** trimer does not (Figure 2 A/C). This results in an overall *C*_3*v*_ symmetry for **2‐Ta**.

**Figure 2:**
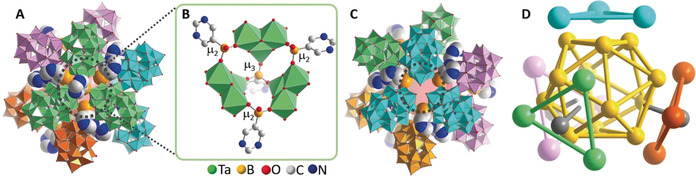
Illustration of **2‐Ta**: **A**: view of the complete nanocapsule, separated into four trimeric **{Ta_3_W_15_}_3_** units (light green, light pink, light blue, orange); **B**: detailed view of the boronic acid (5‐PymB) linkage of the trimers; note the three peripheral μ_2_‐bridging 5‐PymBH units and one central μ_3_‐bridging 5‐PymB unit; **C**: view of the light blue trimer (along the *C*_3_ axis), where the central, μ_3_‐bridging 5‐PymB unit is missing (black dashed circle); **D**: schematic illustration of **2‐Ta**: the twelve μ_2_‐bridging boronic acid B atoms form a icosahedron (yellow), four trigonal faces are capped by POM trimers (light green, light pink, light blue, orange); three of the trimers feature a central μ_3_‐bridging boronic acid (gray), while one trimer (light blue) does not. Hydrogen atoms omitted for clarity.

**2‐Ta** can be rationalized as shown in Figure 2 D: the twelve μ_2_‐bridging boronic acid boron atoms form an idealized icosahedron (yellow). Four icosahedron faces are capped by **{Ta_3_W_15_}**_3_ trimers (light blue, light pink, light green, orange) in a tetrahedral fashion. Three of the trimers feature central μ_3_‐bridging boronic acids (gray) while the fourth trimer (light blue) does not. Note that ^31^P‐NMR spectroscopy of **1** and **2** (see Supporting Information) indicates the stability of the organo‐functionalized clusters in aqueous solution, and further studies on the solution dynamics and stability of these compounds are underway.

To gain insights into the formation mechanism of the organo‐boron functionalized POMs, we carried out a combined experimental and theoretical study. This is based on our hypothesis that the presence of nucleophilic metal oxo sites (here: Ta−O and Nb−O) is required for successful boronic acid linkage. To this end, we performed density functional theory (DFT) calculations using the Amsterdam Density Functional (ADF) package^35^ to compare the electronic structures of the primary POM building unit **{M_3_W_15_}**. To rationalize how different group 5 metals affect the Mulliken charge distribution, we compared the species **{Ta_3_W_15_}**, **{Nb_3_W_15_}** and **{V_3_W_15_}**.

As shown in Figure 3, the three species show significantly different Mulliken charge distribution, and interpretation of the negative charge density of the terminal (O_t_(M)) and bridging (O_b_(M)) oxo groups in the three polyanions indicate a Lewis basicity sequence of O(Ta) > O(Nb) > O(V). This is in line with initial experimental observations which show that the tantalum‐based species **1‐Ta** and **2‐Ta** are formed at higher pH (pH 4.0–0.0) compared with the Nb‐species **1‐Nb** and **2‐Nb**, which require lower pH values of pH 1.0‐0.0 to be formed (see Supporting Information). We propose that this is due to the different reactivity of the Ta−O vs. Nb−O groups, so that formation of the M−O−B linkages is facilitated by the higher basicity/nucleophilicity of the Ta−O bonds compared with the Nb−O bonds. Further evidence is provided by the fact that the V‐based species **{V_3_W_15_}** does not show reactivity towards various boronic acids, and we were not able to identify or isolate any boronic‐acid‐functionalized products from these reactions, even at low pH values.

**Figure 3:**
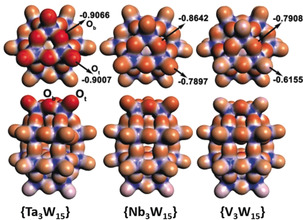
Molecular Electrostatic Potentials (MEPs) of **{M_3_W_15_}** (M=Ta, Nb, V) in top view (top) and the side view (bottom) and the calculated Mulliken charges of the terminal (O_t_) and bridging (O_b_) oxo ligands based on DFT calculations.

In summary, we report a supramolecular organo‐functionalization strategy which allows the boronic‐acid‐based linkage of Dawson polyoxotungstate anions into tetrameric and dodecameric nanocapsules. The synthetic principle uses the selective reactivity between the aromatic boronic acids and the Ta‐ and Nb‐functionalized sites of the Dawson anions, so that controlled aggregation of the species into supramolecular capsules rather than infinite networks is possible. Initial mechanistic studies allowed us to propose the types of POMs which are suitable for boronic acid functionalization. The principles reported could lead to a new class or POM‐based organo‐functionalized architectures for confinement‐controlled catalysis, supramolecular separation and substrate transport.

## Conflict of interest

The authors declare no conflict of interest.

## Supporting information

As a service to our authors and readers, this journal provides supporting information supplied by the authors. Such materials are peer reviewed and may be re‐organized for online delivery, but are not copy‐edited or typeset. Technical support issues arising from supporting information (other than missing files) should be addressed to the authors.

Supplementary
