# Supplementary material for: Organoboron‐Functionalization Enables the Hierarchical Assembly of Giant Polyoxometalate Nanocapsules
Source: Angew Chem Int Ed Engl. 2020 Apr 8;59(22):8537–40. doi: 10.1002/anie.202003550 (PMC7318661; doi:10.1002/anie.202003550)
Supplement: Supplementary file 1 — Supplementary [file ANIE-59-8537-s001.pdf]

Angewandte Chemie
Eine Zeitschrift der Gesellschaft Deutscher Chemiker

Supporting Information

Organoboron-Functionalization Enables the Hierarchical Assembly of Giant Polyoxometalate Nanocapsules

Shujun Li, Yanfang Zhou, Nana Ma, Jie Zhang, Zhiping Zheng, Carsten Streb,* and Xuenian Chen**

anie_202003550_sm_miscellaneous_information.pdf

Supporting Information

Table of Contents

S1 Experimental procedures 2-3
S2 Detailed structural illustrations .... 3−4
S3 X-ray crystallography .... 4−5
S4 ^{31}P NMR spectra .... 6−7
S5 FTIR spectroscopy and Thermal analyses .... 8−10
S6 Theoretical calculation .... 10−12
S7 References 13
S8 Author contributions 13

S1 Experimental procedures

1.1 Materials and Instruments

The precursors $K_8H[P_2W_{15}(NbO_2)_3O_{59}]\cdot 12H_2O$ and $K_5Na_4[P_2W_{15}O_{59}(TaO_2)_3]\cdot 17H_2O$ were synthesized according to the procedure described in the literature.[1,2] All other reagents were readily available from commercial sources and used without further purification. The FTIR spectra in KBr pellets were recorded in the range 400–4000 cm^{-1} with a VECTOR 22 Bruker spectrophotometer at room temperature. Elemental analyses for B, Na, P, Eu, K, Nb, W and Ta were determined with a PLASMASPEC (I) ICP atomic emission spectrometer. Elemental analyses for B, C and N were performed on a Perkin-Elmer 2400 elemental analyzer. The thermal behaviors of the title compounds were examined by synchronous thermal analyses (TG, Netzsch 449C). The samples were heated to 1000 °C with a heating rate of 5 °C /min, under a flowing N_2 atmosphere. ^{31}P NMR spectra were recorded on a Bruker Advance 400 or 600 MHz spectrometer. Chemical shift values of ^{31}P NMR spectra were referenced externally to 85% H_3PO_4.

1.2 Synthesis of the title compounds

Synthesis of $Na_4H_{24}[(3\text{-PyB})_4(P_2W_{15}Ta_3O_{62})_4]\cdot 7C_5H_5N\cdot 45H_2O$ (**1-Ta**): A sample of $K_5Na_4[P_2W_{15}O_{59}(TaO_2)_3]\cdot 17H_2O$ (0.20 g, 0.04 mmol) was dissolved in 25 mL of deionized water at 75 °C. Solid $NaHSO_3$ (0.04 g, 0.38 mmol) was added with stirring until the yellow solution became colorless. Then, 3-PyB(OH)$_2$ (0.01 g, 0.081 mmol) and $EuCl_3\cdot 6H_2O$ (0.02 g, 0.055 mmol) were added respectively. The pH of the resulting solution was adjusted to 2.0 with hydrochloric acid (1.0 M) and the mixture was further stirred at 75 °C for 20 min. After that the reaction solution was filtrated and left for evaporation. Yellowish polyhedral crystals were obtained within two weeks. Yield: 0.13 g (67% based on $K_5Na_4[P_2W_{15}O_{59}(TaO_2)_3]\cdot 17H_2O$). Anal. Calcd (%): B 0.22, C 3.43, N 0.80, Na 0.48, P 1.28, Ta 11.28, W 57.29; found B 0.21, C 3.46, N 0.82, Na 0.50, P 1.30, Ta 11.23, W 57.14. IR (KBr disks): 1626 (w), 1460 (vw), 1400 (vw), 1351 (vw), 1206 (vw), 1089 (s), 951 (s), 904 (m), 758 (vs), 523 (w) cm^{-1}. $^{31}P\{^1H\}$ NMR (161 MHz, D_2O) δ = −11.7 (s), −13.3 (s).

Synthesis of $Na_2H_{26}[(3\text{-PyB})_4(P_2W_{15}Nb_3O_{62})_4]\cdot 2C_5H_5N\cdot 37H_2O$ (**1-Nb**): A sample of $K_8H[P_2W_{15}(NbO_2)_3O_{59}]\cdot 12H_2O$ (0.20 g, 0.04 mmol) was dissolved in 25 mL of deionized water at 75 °C. Solid $NaHSO_3$ (0.04 g, 0.38 mmol) was added with stirring until the yellow solution became colorless. Then, 3-PyB(OH)$_2$ (0.01 g, 0.081 mmol) were added. The pH of the resulting solution was adjusted to 1.0 with hydrochloric acid (1 M) and the mixture was further stirred at 75 °C for 20 min. After that the reaction solution was filtrated and left for evaporation. Yellowish polyhedral crystals were obtained within two weeks. Yield: 0.13 g (72% based on $K_8H[P_2W_{15}(NbO_2)_3O_{59}]\cdot 12H_2O$). Anal. Calcd (%): B 0.24, C 2.03, N 0.47, Na 0.26, P 1.40, Nb 6.28, W 62.17; found B 0.23, C 2.01, N 0.49, Na 0.28, P 1.39, Nb 6.32, W 62.21. IR (KBr disks): 1620 (w), 1461 (vw), 1402 (vw), 1351 (vw), 1203 (vw), 1093 (s), 958 (m), 898 (m), 749 (vs), 526(w) cm^{-1}. $^{31}P\{^1H\}$ NMR (161 MHz, D_2O) δ = −8.0 (s), −13.5 (s).

Synthesis of $Eu_3Na_{20.5}K_{2.5}H_{54}[K_4(P_2W_{15}Ta_3O_{62})_{12}(5\text{-PymB})_3(5\text{-PymBOH})_{12}]\cdot 94H_2O$ (**2-Ta**): A sample of $K_5Na_4[P_2W_{15}O_{59}(TaO_2)_3]\cdot 17H_2O$ (0.20 g, 0.04 mmol) was dissolved in 25 mL of deionized water at 75 °C. Solid $NaHSO_3$ (0.04 g, 0.38 mmol) was added with stirring until the yellow solution became colorless. Then, 5-PymB(OH)$_2$ (0.01 g, 0.081 mmol) and $EuCl_3\cdot 6H_2O$ (0.02 g, 0.055mmol) were added respectively. The pH of the resulting solution was adjusted to 2.0 with hydrochloric acid (1.0 M), and the mixture was further stirred at 75 °C for 20 min. After that the reaction solution was filtrated and left for evaporation. Yellowish polyhedral crystals were obtained within two weeks. Yield: 0.15 g (79% based on $K_5Na_4[P_2W_{15}O_{59}(TaO_2)_3]\cdot 17H_2O$). Anal. Calcd (%): B 0.28, C 1.27, N 0.74, Na 0.74, P 1.31, K 0.45, Eu 0.80, Ta 11.48, W 58.32; found B 0.27, C 1.25, N 0.76, Na 0.76, P 1.33 K 0.46, Eu 0.80, Ta 11.51, W 58.48. IR (KBr disks): 1613 (w), 1404 (vw), 1094 (s), 956 (m), 908 (m), 767 (vs), 522 (w) cm^{-1}. $^{31}P\{^1H\}$ NMR (161 MHz, D_2O) δ = -11.5 (s), -13.1 (s).

Synthesis of $Na_{15}H_{71}[K_4(P_2W_{15}Nb_3O_{62})_{12}(5\text{-PymB})_3(5\text{-PymBOH})_{12}]\cdot 99H_2O$ (**2-Nb**): A sample of $K_8H[P_2W_{15}(NbO_2)_3O_{59}]\cdot 12H_2O$ (0.20 g, 0.04 mmol) was dissolved in 25 mL of deionized water at 75 °C. Solid $NaHSO_3$ (0.04 g, 0.38 mmol) was added with stirring until the yellow solution became colorless. Then, 5-PymB(OH)$_2$ (0.01 g, 0.081 mmol) and $EuCl_3\cdot 6H_2O$ (0.02 g, 0.055 mmol) were added. The pH of the resulting solution was adjusted to 1.0 with hydrochloric acid (1.0 M), and the mixture was stirred at 75 °C for 20 min. After that the reaction solution was filtered and left for evaporation. Yellowish polyhedral crystals were obtained within two weeks. Yield: 0.14 g (73% based on $K_8H[P_2W_{15}(NbO_2)_3O_{59}]\cdot 12H_2O$). Anal. Calcd (%): B 0.31, C 1.36, N 0.79, Na 0.65, P 1.40, K 0.30,Nb 6.31, W 62.46; found B 0.30, C 1.38, N 0.81, Na 0.69, P 1.41, K 0.32, Nb 6.26, W 62.62. IR (KBr disks): 1616 (w), 1427 (vw), 1404 (vw), 1342 (vw), 1180 (m), 1089 (vs), 953 (m), 905 (m), 770 (vs), 526 (w) 486 (w) cm^{-1}. $^{31}P\{^1H\}$ NMR (161 MHz, D_2O) δ = -8.7 (s), -13.7 (s).

It is worth noting that all the title compounds can be synthesized by using peroxy-free monomer $[P_2W_{15}Ta_3O_{62}]^{9-}$ or $[P_2W_{15}Nb_3O_{62}]^{9-}$ as starting precursor under the same reaction conditions without use of $NaHSO_3$. Besides, they can be synthesized in the presence or absence of rare earth ions (La^{3+}, Eu^{3+}, Er^{3+} were demonstrated to work), however, higher yields are observed in the presence of rare earth cations. The reactions of $[P_2W_{15}Ta_3O_{62}]^{9-}$ with 3-PyBH$_2$ or 5-PymBH$_2$ can produce **1-Ta** or **2-Ta** in the pH range of 4.0−0.0 (using aqueous hydrochloric acid). While, similar reactions for $[P_2W_{15}Nb_3O_{62}]^{9-}$ can be achieved only under stronger acidic conditions (pH 1.0−0.0). The higher reactivity of Ta-POMs may be caused by the higher basicity/nucleophilicity of the O_t(Ta) than O_t(Nb) as discussed in the main text. Also note that for charge balance reasons, partial protonation of the clusters was proposed based on the elemental analyses and thermal

analyses; however, note that direct location of the protonation sites by X-ray crystallography has not been possible due to the large number of heavy atoms and general crystal quality.

S2 Detailed structural illustrations

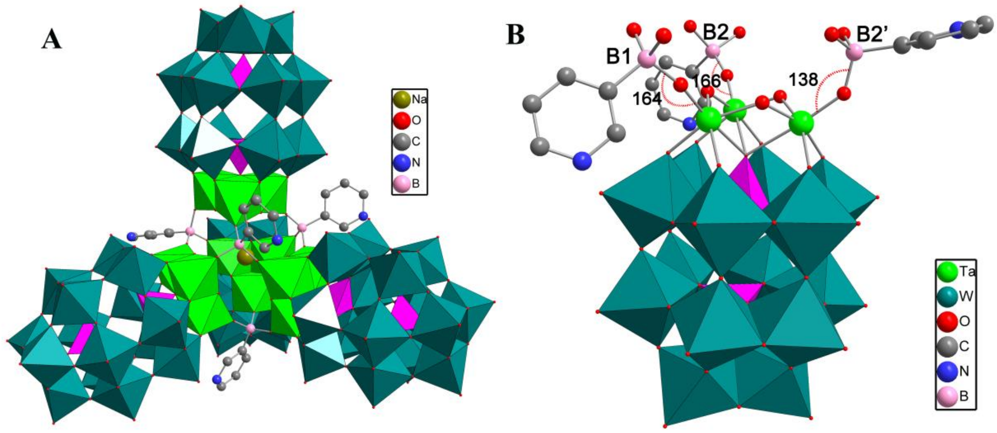

Figure S1. Combined polyhedral/ball-and-stick representation of polyanion **1-Ta** (A) and the coordination environment of each **{Ta_3W_{15}}** in **1-Ta** (B). Each **{Ta_3W_{15}}** unit coordinates to three boronic acid molecules, two of which (B1 and B2) are perpendicular to the **{Ta_3W_{15}}** with average B-O-Ta bond angle of 165°, and the third boronic acid molecule (B2') is pointing away from **{Ta_3W_{15}}** with B-O-Ta bond angle of 138°.

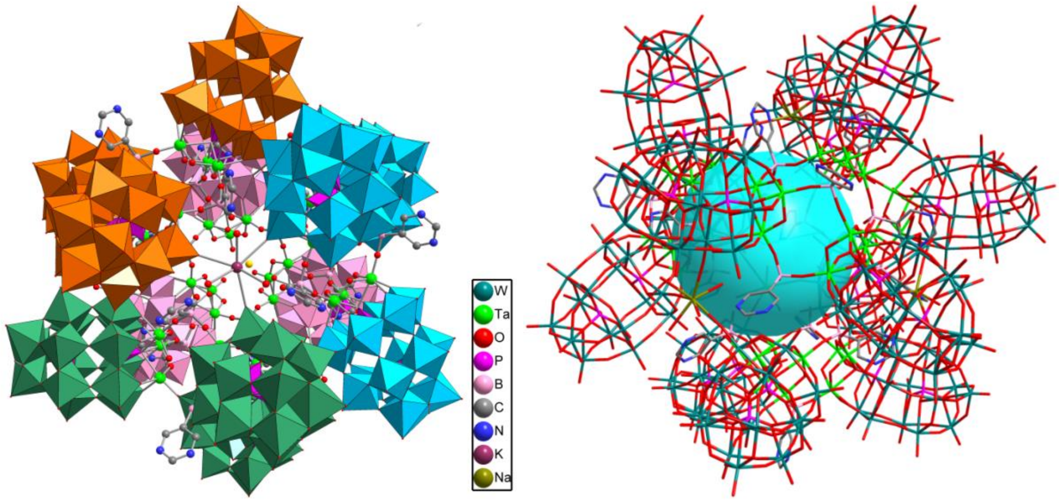

Figure S2. Illustration of **2-Ta** (left) and representation of the central void (right, blue sphere, diameter 1.3 nm).

Note that compound **2-Ta** is linked into larger aggregates by external Na^+ cations as illustrated below:

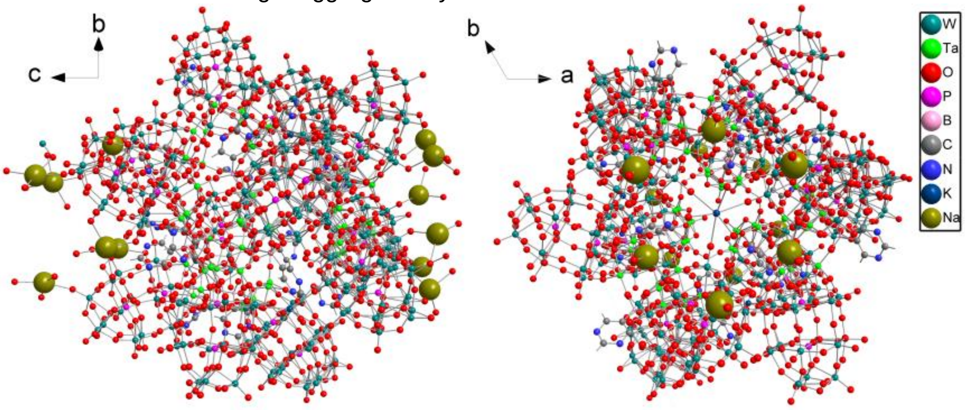

Figure S3. Binding of the external Na^+ linkages at the two ends of the nanocapsule **2-Ta**, viewed along the crystallographic *a* axis (left) and *c* axis (right).

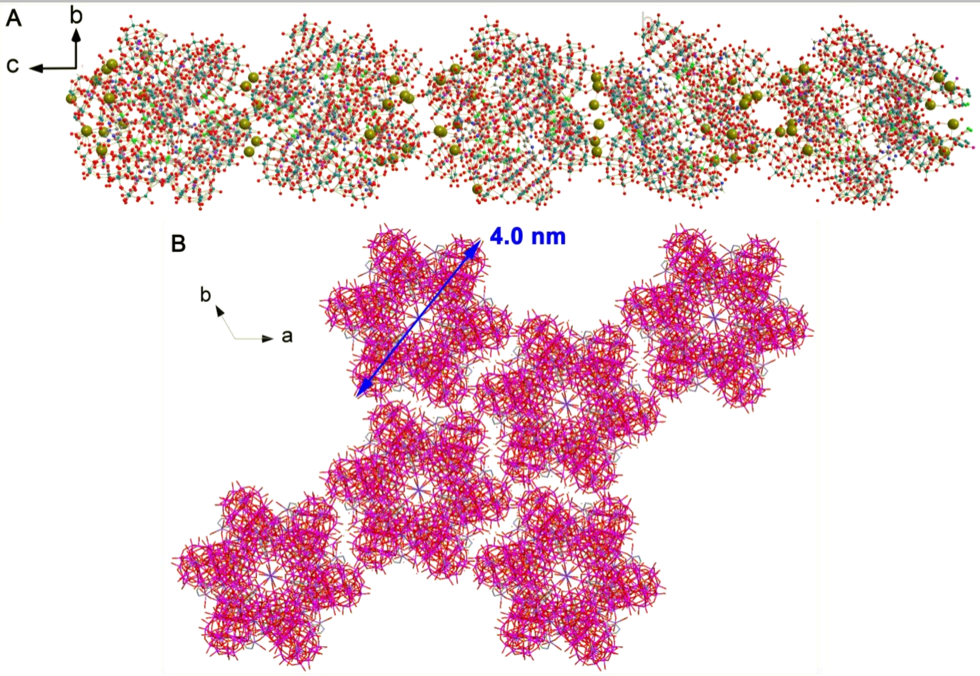

Figure S4. Illustration of the aggregation of individual **2-Ta** nanocapsules by Na^+ linkage, viewed along the crystallographic *a* axis (A) and *c* axis (B). Two neighboring nanocapsules are linked by six Na^+ cations, resulting in a 1D chain along the *c*-axis. The chains stack in the *a*-*b* plane *via* hydrogen-bonding interactions.

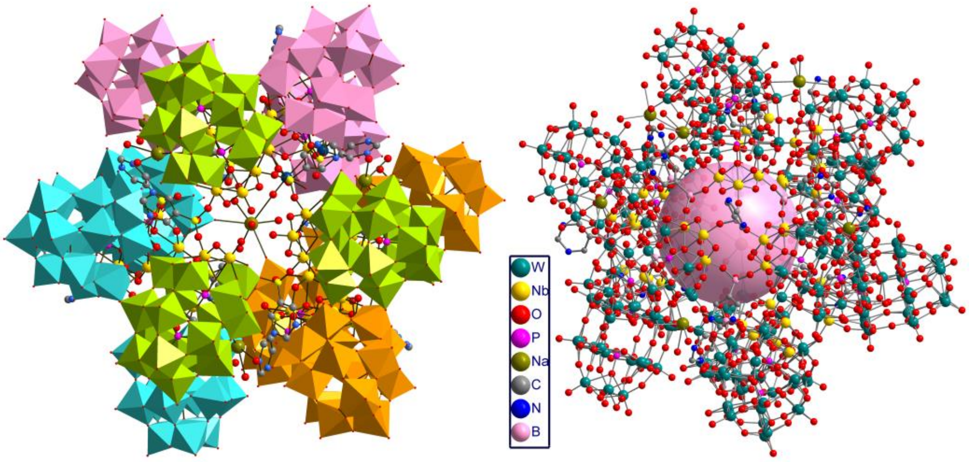

Figure S5. Polyhedral/ball-and-stick representation (left) and the ball-and-stick representation (right) of polyanion **2-Nb** (isostructural with **2-Ta**, only Ta atoms are replaced with Nb atoms).

S3 X-ray Crystallography

Single crystal XRD analysis of **1-Ta**, **1-Nb**, **2-Ta** and **2-Nb** were recorded on a SuperNova Dual diffractometer using graphite-monochromated Cu *Kα* radiation, λ = 1.54184 Å. The linear absorption coefficients, scattering factors for the atoms, and anomalous dispersion corrections were taken from the International Tables for X-Ray Crystallography. Empirical absorption corrections were applied. Structures were solved using direct methods (SHELXT)[3] and refined by full-matrix least-squares (SHELXL) interfaced with the programme $OLEX_2$.[4] Anisotropic thermal parameters were used to refine all non-hydrogen atoms, with the exception for a few oxygen atoms. Those hydrogen atoms attached to lattice water molecules were not located. Crystallization water molecules and guest molecules were estimated by thermogravimetry and only partial guest molecules were achieved with the X-ray structure analysis. The crystal data and structure refinement results are summarized in Table S1. Further details on the crystal structure investigations can be obtained free of charge from The Cambridge Crystallographic Data Centre via https://www.ccdc.cam.ac.uk/structures/ by quoting the depository numbers CCDC-1581190 (**1-Ta**), CCDC-1581189 (**1-Nb**), CCDC-1833025 (**2-Ta**), CCDC-1833026 (**2-Nb**).

Table S1. Crystal data and structural refinements.

Compounds	**1-Ta**	**1-Nb**	**2-Ta**	**2-Nb**
Formula	$H_{165}B_4C_{55}N_{11}O_{293}Na_4P_8Ta_{12}W_{60}$	$H_{132}B_4C_{30}N_6O_{285}Na_2P_8Nb_{12}W_{60}$	$H_{299}B_{15}C_{60}N_{30}O_{850}Na_{20.5}P_{24}K_{6.5}Eu_3Ta_{36}W_{180}$	$H_{314}B_{15}C_{60}N_{30}O_{855}Na_{15}P_{24}K_4Nb_{36}W_{180}$
Formula weight ($g \cdot mol^{-1}$)	19253.56	17619.5	56663.81	53361.92
T (K)	100 K	100 K	100 K	100 K
Wavelength Cu-Kα (Å)	1.54184	1.54184	1.54184	1.54184
Crystal system	monoclinic	monoclinic	trigonal	trigonal
Space group	I2/a	I2/a	R-3	R-3
a (Å)	26.9889(4)	26.8824(4)	57.1169(7)	57.3040(8)
b (Å)	49.6651(5)	49.0974(7)	57.1169(7)	57.3040(8)
c (Å)	32.2254(4)	32.4146(4)	65.1285(8)	65.4141(10)
α(°)	90	90	90	90
β(°)	105.7122(16)	105.6580(10)	90	90
γ(°)	90	90	120	120
V(Å^3)	41581.1(10)	41194.9(10)	184005(5)	186025(6)
Z	4	4	18	18
D_{calc}(mg m^{-3})	3.006	2.805	3.107	2.872
μ(mm^{-1})	36.416	33.675	38.388	33.624
F(000)	32562.0	30240.0	148717.0	140351.0
Crystalsize(mm)	0.12×0.09×0.07	0.1×0.1×0.09	0.24× 0.12 × 0.1	0.12× 0.10 ×0.1
Goodness−of−fit on F^2	1.089	1.099	1.037	1.011
Final *R* indices [I>2σ(I)][a]	R1 = 0.0725<br>wR2 = 0.2138	R1 = 0.0809<br>wR2 = 0.2328	R1 = 0.1005<br>wR2 = 0.2649	R1 = 0.1013<br>wR2 = 0.2315
R indices[a] (all data)	R1 = 0.1000<br>wR2 = 0.2300	R1 = 0.1135<br>wR2 =0.2544	R1 = 0.1875<br>wR2 = 0.3016	R1 = 0.1893<br>wR2 =0.3022

[a] $R_1 = \sum||F_o|-|F_c||/\sum|F_o|$; $wR_2 = \{\sum[w(F_o^2-F_c^2)^2]/\sum[w(F_o^2)^2]\}^{1/2}$

S4 ^{31}P NMR spectra

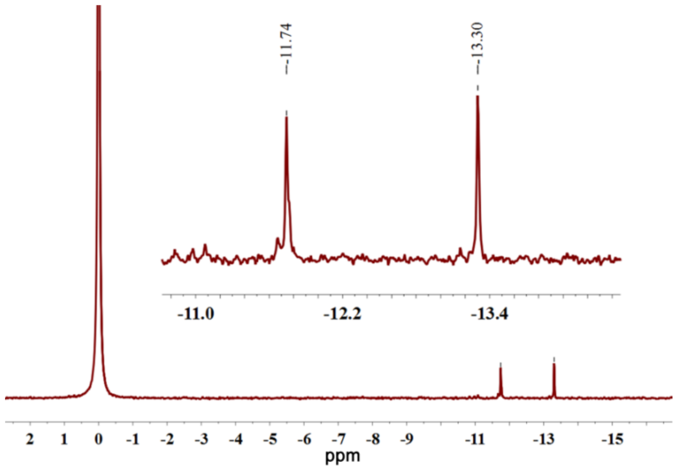

Figure S6. ^{31}P NMR spectrum of **1-Ta**. As-synthesized sample dissolved in DCl/D_2O solution with final pD of 2.0.

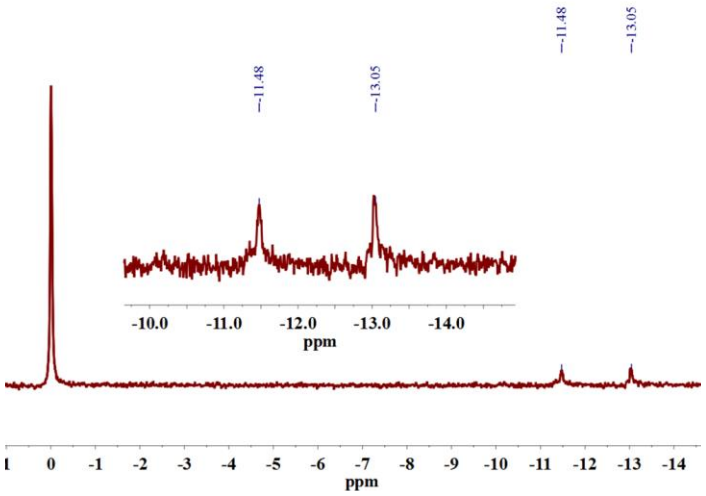

Figure S7. ^{31}P NMR spectrum of **2-Ta**. As-synthesized sample dissolved in DCl/D_2O solution with final pD of 2.0.

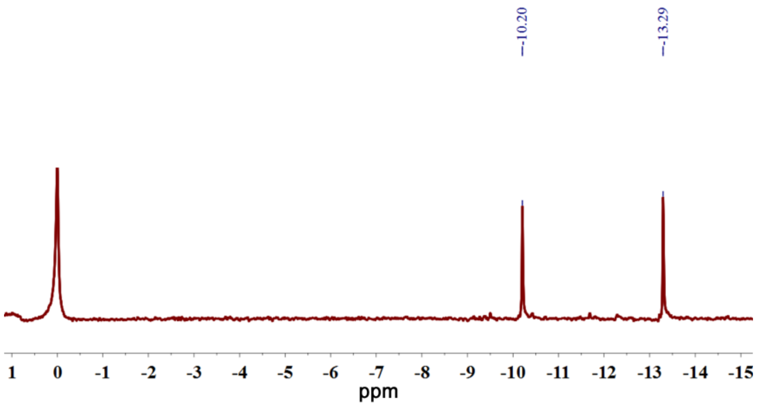

Figure S8. ^{31}P NMR spectrum of $[P_8W_{60}Ta_{12}(H_2O)_4(OH)_8O_{236}]^{20-}$. As-synthesized sample dissolved in DCl/D_2O solution ($[D^+]$ = 0.5 M).

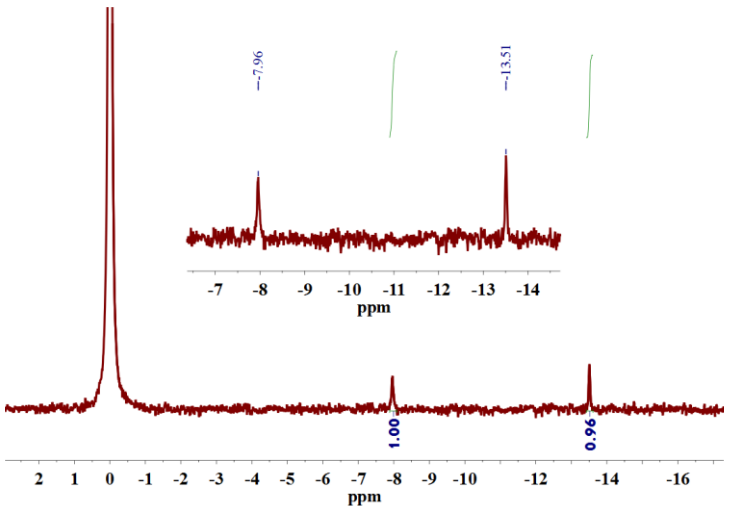

Figure S9. ^{31}P NMR spectrum of **1-Nb**. As-synthesized sample dissolved in DCl/D_2O solution with final pD of 1.0.

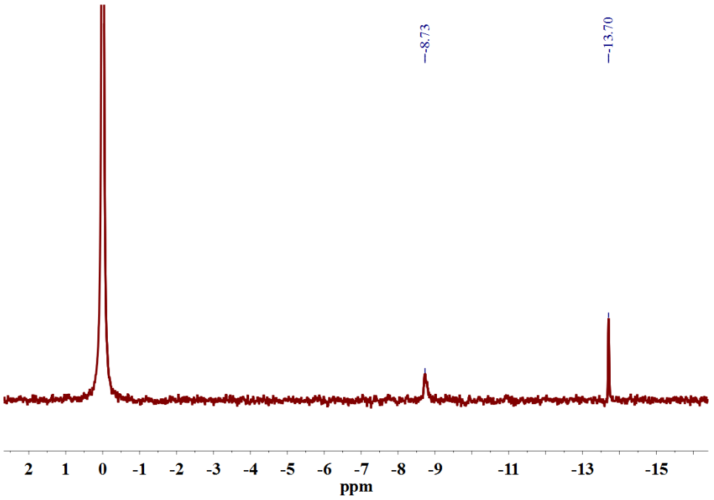

Figure S10. ^{31}P NMR spectrum of **2-Nb**. As-synthesized sample dissolved in DCl/D_2O solution with final pD of 1.0.

Table S2. The ^{31}P NMR chemical shift of chemical shifts of $\{P_2W_{15}Ta_3O_{62}\}$ derivatives.

Compounds	chemical shifts (ppm)[a]
**1-Ta**	-11.7, -13.3
**2-Ta**	-11.5, -13.1
**$[P_8W_{60}Ta_{12}(H_2O)_4(OH)_8O_{236}]^{20-}$** (Ta-reference compound)	-10.2, -13.3
**1-Nb**	−8.0, −13.5
**2-Nb**	−8.7, −13.7
**$Nb_3P_2W_{15}$** (Nb reference compound)	−7.2, −13.8

[a] Chemical shift values ^{31}P NMR spectra were referenced externally to 85% H_3PO_4 (δ 0).

S5 FTIR spectroscopy and Thermal analyses

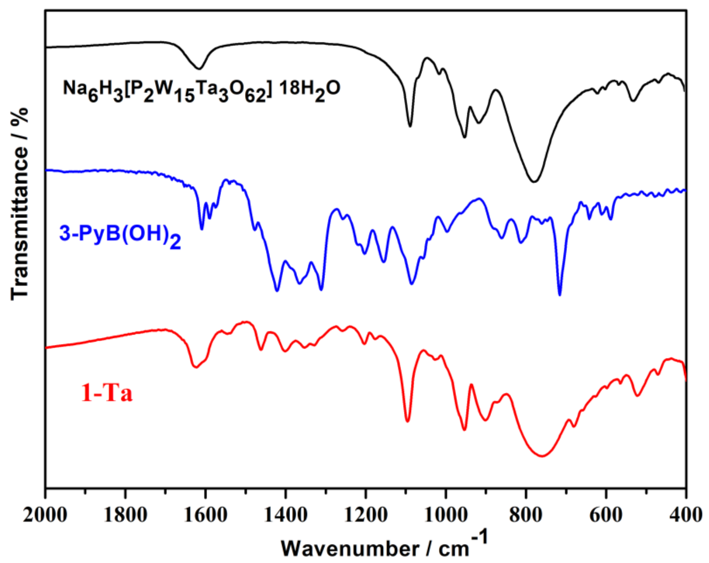

Figure S11. IR spectra of **1-Ta** and precursors.

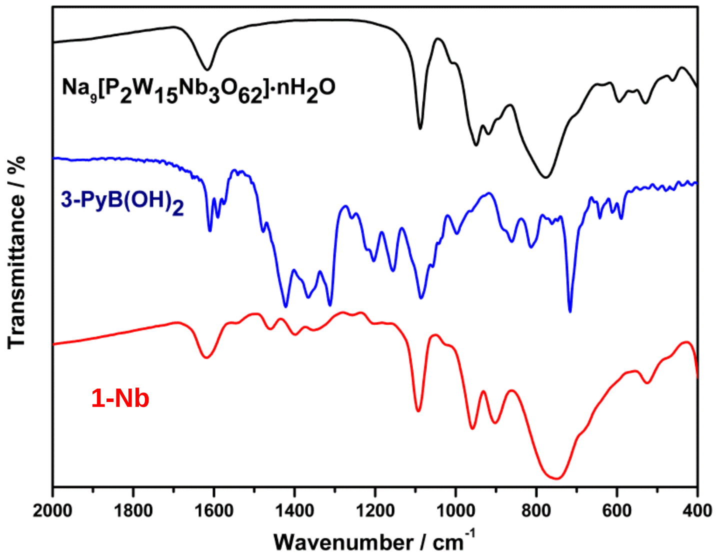

Figure S12. IR spectra of **1-Nb** and precursors.

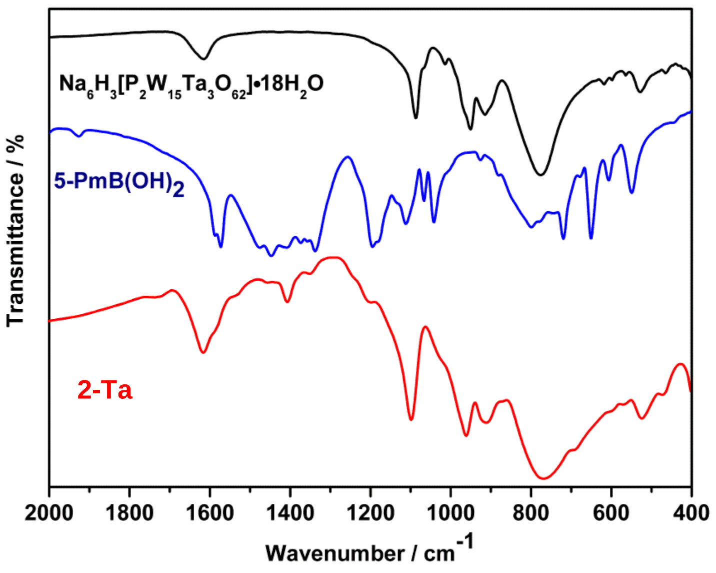

Figure S13. IR spectra of **2-Ta** and precursors.

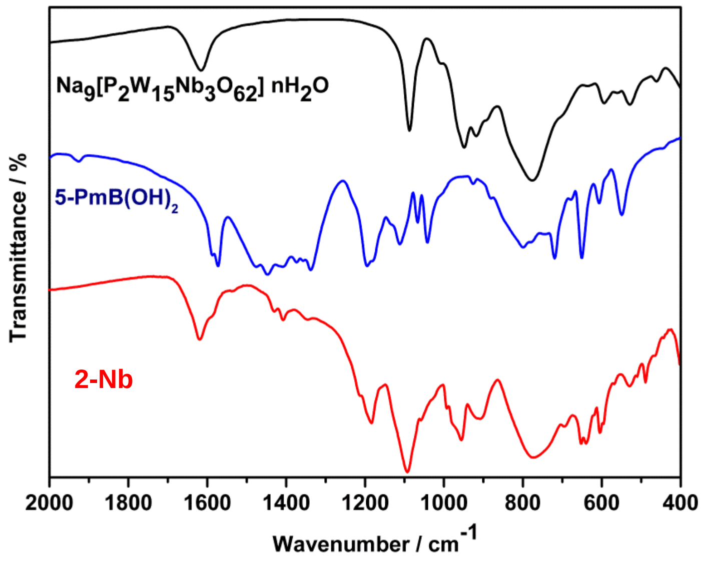

Figure S14. IR spectra of **2-Nb** and precursors.

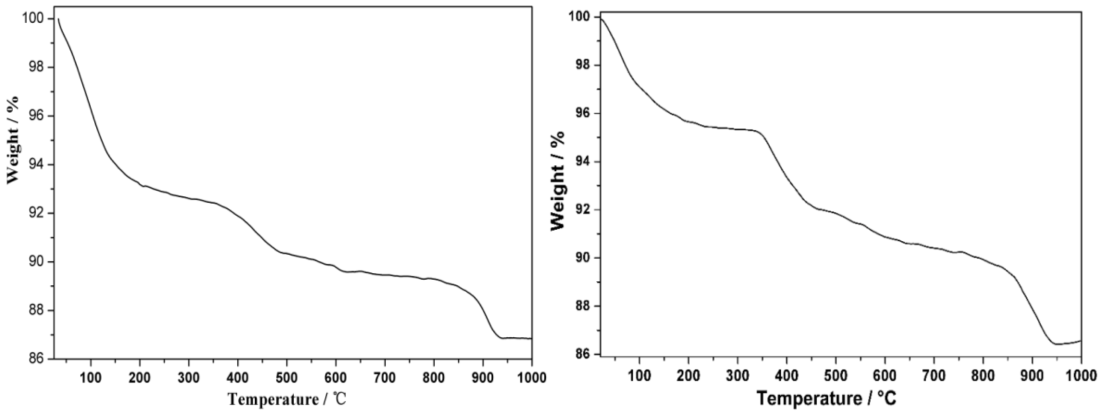

Figure S15. TG curves of **1-Ta** (left) and **1-Nb** (right).

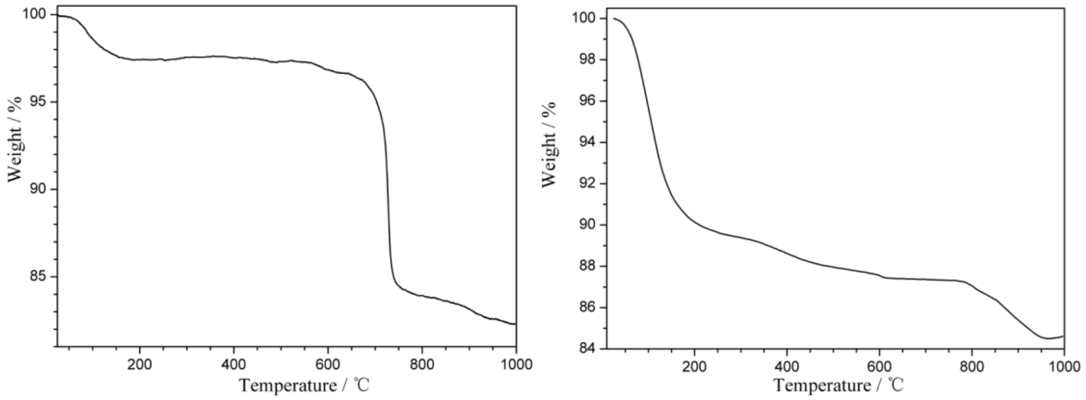

Figure S16. TG curves of **2-Ta** (left) and **2-Nb** (right).

S6 Theoretical calculations

Computational details: All the DFT calculations were performed using the ADF package.[5-7] The geometry optimizations employed the BP86[8,9] generalized gradient approximations (GGA). Triple-ξ plus polarization basis sets (TZP) were used to describe the valence electrons of all atoms, large frozen core was described by means of single Slater functions. The zero-order regular approximation (ZORA) was adopted in all the calculations to account for the scalar relativistic effects.[10] The solvent effects were employed in the geometry optimization by using a conductor-like screening model (COSMO)[11] of solvation with the solvent-excluding-surface. The solute dielectric constant of water was set to 78.39. The van der Waals radii for the atoms in POM, which actually define the cavity in the COSMO, are 1.40, 1.92, 2.10, 2.07, and 2.17 for O, P, Nb, W and Ta respectively.

Cartesian coordinates

$P_2W_{15}Ta_3^{9-}$

	x	y	z
O	0.190502	-2.973399	3.663884
O	-0.818759	1.418133	5.780150
O	-2.670291	-1.321720	3.663884
O	-4.632607	2.460210	-2.252019
O	-2.670291	1.321720	3.663884
O	-2.600180	1.303222	-3.741032
O	-0.818759	-1.418133	5.780150
O	-0.770241	-1.334097	-5.719056
O	-2.600180	-1.303222	-3.741032
O	-0.770241	1.334097	-5.719056
O	-2.999330	2.122853	-0.049985
O	1.620214	-2.806294	-6.183308
O	-2.999330	-2.122853	-0.049985
O	0.171467	-2.903433	-3.741032
O	-0.338780	-3.658923	-0.049985
O	-2.001761	-3.467153	-2.210876
O	-0.738323	-1.278812	-1.574824
O	1.683825	-2.916471	-1.625261
O	-0.737550	-1.277474	1.562358
O	1.680044	-2.909921	1.491592
O	0.000000	0.000000	3.627479
O	-3.360087	0.000000	1.491592
O	-2.001761	3.467153	-2.210876
O	-4.645476	2.463290	2.140140
O	-0.737550	1.277474	1.562358
O	-0.738323	1.278812	-1.574824
O	-3.367651	0.000000	-1.625261
O	-4.632607	-2.460210	-2.252019
O	0.000000	0.000000	-3.623667
O	-3.240429	0.000000	-6.183308
O	0.185699	-5.242060	-2.252019
O	0.189467	-5.254745	2.140140
O	1.671217	-2.894633	6.233183
O	-3.342435	0.000000	6.233183
O	-2.005998	3.474490	2.079732
O	-2.005998	-3.474490	2.079732
O	-4.645476	-2.463290	2.140140
P	0.000000	0.000000	2.049774
P	0.000000	0.000000	-2.034977
Ta	-1.981474	0.000000	5.070701
Ta	0.990737	-1.716007	5.070701
W	-3.033263	1.841014	1.918364
W	-3.033263	-1.841014	1.918364
W	-0.077734	-3.547390	1.918364
W	-3.036289	-1.864976	-1.897726
W	-0.096972	-3.561991	-1.897726
W	0.991964	-1.718132	-4.978472
W	-1.983928	0.000000	-4.978472
W	-3.036289	1.864976	-1.897726
O	2.479788	-1.651680	3.663884
O	2.479788	1.651680	3.663884
O	0.185699	5.242060	-2.252019
O	0.190502	2.973399	3.663884
O	0.171467	2.903433	-3.741032
O	1.637519	0.000000	5.780150
O	1.540483	0.000000	-5.719056
O	2.428713	1.600211	-3.741032
O	-0.338780	3.658923	-0.049985
O	3.338110	1.536069	-0.049985

O	2.428713	-1.600211	-3.741032
O	3.338110	-1.536069	-0.049985
O	4.003523	0.000000	-2.210876
O	1.476645	0.000000	-1.574824
O	1.475100	0.000000	1.562358
O	1.680044	2.909921	1.491592
O	0.189467	5.254745	2.140140
O	1.683825	2.916471	-1.625261
O	4.446908	2.781850	-2.252019
O	1.620214	2.806294	-6.183308
O	4.446908	-2.781850	-2.252019
O	4.456010	-2.791456	2.140140
O	1.671217	2.894633	6.233183
O	4.011996	0.000000	2.079732
O	4.456010	2.791456	2.140140
Ta	0.990737	1.716007	5.070701
W	-0.077734	3.547390	1.918364
W	3.110997	1.706376	1.918364
W	3.110997	-1.706376	1.918364
W	3.133261	1.697016	-1.897726
W	3.133261	-1.697016	-1.897726
W	0.991964	1.718132	-4.978472
W	-0.096972	3.561991	-1.897726

$P_2W_{15}Nb_3^{9-}$

	x	y	z
O	0.188594	-2.987052	3.668278
O	-0.816251	1.413788	5.799785
O	-2.681160	-1.330199	3.668278
O	-4.633510	2.470836	-2.255378
O	-2.681160	1.330199	3.668278
O	-2.608172	1.306943	-3.752050
O	-0.816251	-1.413788	5.799785
O	-0.770171	-1.333975	-5.726746
O	-2.608172	-1.306943	-3.752050
O	-0.770171	1.333975	-5.726746
O	-2.998776	2.121265	-0.055506
O	1.617486	-2.801568	-6.199524
O	-2.998776	-2.121265	-0.055506
O	0.172241	-2.912215	-3.752050
O	-0.337681	-3.657649	-0.055506
O	-2.002356	-3.468182	-2.216433
O	-0.740895	-1.283267	-1.586554
O	1.688391	-2.924378	-1.634536
O	-0.739989	-1.281699	1.570576
O	1.686332	-2.920812	1.494051
O	0.000000	0.000000	3.634550
O	-3.372664	0.000000	1.494051
O	-2.002356	3.468182	-2.216433
O	-4.644906	2.476839	2.132402
O	-0.739989	1.281699	1.570576
O	-0.740895	1.283267	-1.586554
O	-3.376781	0.000000	-1.634536
O	-4.633510	-2.470836	-2.255378
O	0.000000	0.000000	-3.632092
O	-3.234972	0.000000	-6.199524
O	0.176948	-5.248155	-2.255378
O	0.177448	-5.261026	2.132402
O	1.669265	-2.891252	6.232162
O	-3.338530	0.000000	6.232162
O	-2.007662	3.477372	2.071206
O	-2.007662	-3.477372	2.071206
O	-4.644906	-2.476839	2.132402
P	0.000000	0.000000	2.058014
P	0.000000	0.000000	-2.038379
W	-3.035050	1.840367	1.923039
W	-3.035050	-1.840367	1.923039
W	-0.076280	-3.548614	1.923039
W	-3.039646	-1.865652	-1.901049
W	-0.095879	-3.565236	-1.901049
W	0.993428	-1.720668	-4.984102
W	-1.986856	0.000000	-4.984102
W	-3.039646	1.865652	-1.901049
O	2.492566	-1.656853	3.668278
O	2.492566	1.656853	3.668278
O	0.176948	5.248155	-2.255378
O	0.188594	2.987052	3.668278
O	0.172241	2.912215	-3.752050
O	1.632502	0.000000	5.799785
O	1.540342	0.000000	-5.726746
O	2.435931	1.605272	-3.752050
O	-0.337681	3.657649	-0.055506
O	3.336457	1.536384	-0.055506
O	2.435931	-1.605272	-3.752050
O	3.336457	-1.536384	-0.055506
O	4.004712	0.000000	-2.216433
O	1.481789	0.000000	-1.586554
O	1.479978	0.000000	1.570576
O	1.686332	2.920812	1.494051
O	0.177448	5.261026	2.132402
O	1.688391	2.924378	-1.634536
O	4.456562	2.777319	-2.255378
O	1.617486	2.801568	-6.199524
O	4.456562	-2.777319	-2.255378
O	4.467459	-2.784187	2.132402
O	1.669265	2.891252	6.232162
O	4.015324	0.000000	2.071206
O	4.467459	2.784187	2.132402
W	-0.076280	3.548614	1.923039
W	3.111329	1.708247	1.923039
W	3.111329	-1.708247	1.923039
W	3.135525	1.699584	-1.901049
W	3.135525	-1.699584	-1.901049
W	0.993428	1.720668	-4.984102
W	-0.095879	3.565236	-1.901049
Nb	0.991910	-1.718039	5.089148
Nb	-1.983821	0.000000	5.089148
Nb	0.991910	1.718039	5.089148

$P_2W_{15}V_3^{9-}$

	x	y	z
O	0.184031	-2.900043	3.651492
O	-0.765074	1.325148	5.644467
O	-2.603527	-1.290646	3.651492
O	-4.634852	2.469772	-2.253472
O	-2.603527	1.290646	3.651492
O	-2.608480	1.307369	-3.745204
O	-0.765074	-1.325148	5.644467
O	-0.770086	-1.333828	-5.719879
O	-2.608480	-1.307369	-3.745204
O	-0.770086	1.333828	-5.719879
O	-3.008965	2.132660	-0.048665
O	1.618315	-2.803004	-6.191206
O	-3.008965	-2.132660	-0.048665
O	0.172025	-2.912694	-3.745204
O	-0.342455	-3.672170	-0.048665
O	-2.002378	-3.468221	-2.213123
O	-0.741355	-1.284065	-1.581660
O	1.688726	-2.924960	-1.626994
O	-0.741268	-1.283915	1.496523
O	1.684140	-2.917017	1.476712
O	0.000000	0.000000	3.545380
O	-3.368281	0.000000	1.476712
O	-2.002378	3.468221	-2.213123
O	-4.625798	2.453614	2.188238
O	-0.741268	1.283915	1.496523
O	-0.741355	1.284065	-1.581660
O	-3.377453	0.000000	-1.626994

O	-4.634852	-2.469772	-2.253472
O	0.000000	0.000000	-3.626064
O	-3.236630	0.000000	-6.191206
O	0.178541	-5.248785	-2.253472
O	0.188007	-5.232866	2.188238
O	1.543997	-2.674281	6.049908
O	-3.087993	0.000000	6.049908
O	-2.007873	3.477738	2.080585
O	-2.007873	-3.477738	2.080585
O	-4.625798	-2.453614	2.188238
P	0.000000	0.000000	1.988612
P	0.000000	0.000000	-2.031817
W	-3.014054	1.827244	1.938204
W	-3.014054	-1.827244	1.938204
W	-0.075412	-3.523870	1.938204
W	-3.041207	-1.867285	-1.888836
W	-0.096513	-3.567405	-1.888836
W	0.993951	-1.721574	-4.974223
W	-1.987902	0.000000	-4.974223
W	-3.041207	1.867285	-1.888836
O	2.419495	-1.609398	3.651492
O	2.419495	1.609398	3.651492
O	0.178541	5.248785	-2.253472
O	0.184031	2.900043	3.651492
O	0.172025	2.912694	-3.745204
O	1.530149	0.000000	5.644467
O	1.540172	0.000000	-5.719879
O	2.436454	1.605326	-3.745204
O	-0.342455	3.672170	-0.048665
O	3.351420	1.539510	-0.048665
O	2.436454	-1.605326	-3.745204
O	3.351420	-1.539510	-0.048665
O	4.004757	0.000000	-2.213123
O	1.482710	0.000000	-1.581660
O	1.482537	0.000000	1.496523
O	1.684140	2.917017	1.476712
O	0.188007	5.232866	2.188238
O	1.688726	2.924960	-1.626994
O	4.456311	2.779013	-2.253472
O	1.618315	2.803004	-6.191206
O	4.456311	-2.779013	-2.253472
O	4.437791	-2.779252	2.188238
O	1.543997	2.674281	6.049908
O	4.015746	0.000000	2.080585
O	4.437791	2.779252	2.188238
W	-0.075412	3.523870	1.938204
W	3.089467	1.696626	1.938204
W	3.089467	-1.696626	1.938204
W	3.137719	1.700120	-1.888836
W	3.137719	-1.700120	-1.888836
W	0.993951	1.721574	-4.974223
W	-0.096513	3.567405	-1.888836
V	0.931787	1.613902	4.964913
V	0.931787	-1.613902	4.964913
V	-1.863573	0.000000	4.964913

S7 References

[1] S. Li, S. Liu, S. Liu, Y. Liu, Q. Tang, Z. Shi, S. Ouyang, J. Ye, *J. Am. Chem. Soc*. **2012**, *134*, 19716–19721.
[2] J. Gong, Y.-G. Chen, L.-Y. Qu, Q. Liu, Polyhedron **1996**, *15*, 2273–2277.
[3] G. M. Sheldrick, *Acta Cryst. A* **2008**, *64*, 112–122.
[4] O. V. Dolomanov, L. J. Bourhis, R. J. Gildea, J. A. K. Howard, H. Puschmann, *J. Appl. Cryst.* **2009**, *42*, 339–341.
[5] ADF2017, SCM, Theoretical Chemistry, Vrije Universiteit, Amster-dam, The Netherlands, http://www.scm.com.
[6] C. F. Guerra, J. G. Snijders, G. T. Velde, E. J. Baerends, *Theor. Chem. Acc.* **1998**, *99*, 391–403.
[7] G. T. Velde, F. M. Bickelhaupt, E. J. Baerends, C. F. Guerra, S. J. A. V. Gisbergen, J. G. Snijders, T. Ziegler, *J. Comput. Chem.* **2001**, *22*, 931–967.
[8] A. D. Becke, *Physical Review A* **1988**, *38*, 3098–3100.
[9] J. P. Perdew, Physical Review B **1986**, *33*, 8822–8824.
[10] E. V. Lenthe, R. V. Leeuwen, E. J. Baerends, J. G. Snijders, *J. Chem. Phys.* **1993**, *99*, 4597–4610.
[11] C. C. Pye, T. Ziegler, *Theor. Chem. Acc.* **1999**, *101*, 396–408.

S8 Author contributions

Z. Zheng and X. Chen conceived the project. C. Streb, X. Chen, Z. Zheng and S. Li supervised the project, designed the experiments and wrote the manuscript. S. Li and Y. Zhou co-worked on the synthesis and characterizations of the title compounds. S. Li and J. Zhang collected the structural data and provided detailed refinements on the crystal structures. C. Streb provided detailed structural analysis. N. Ma performed the DFT calculations. All authors co-wrote the manuscript.
